# Volatile Exchange between Undamaged Plants - a New Mechanism Affecting Insect Orientation in Intercropping

**DOI:** 10.1371/journal.pone.0069431

**Published:** 2013-07-29

**Authors:** Velemir Ninkovic, Iris Dahlin, Andja Vucetic, Olivera Petrovic-Obradovic, Robert Glinwood, Ben Webster

**Affiliations:** 1 Department of Ecology, Swedish University of Agricultural Sciences, Uppsala, Sweden; 2 Faculty of Agriculture, University of Belgrade, Zemun-Belgrade, Serbia; CNRS, France

## Abstract

Changes in plant volatile emission can be induced by exposure to volatiles from neighbouring insect-attacked plants. However, plants are also exposed to volatiles from unattacked neighbours, and the consequences of this have not been explored. We investigated whether volatile exchange between undamaged plants affects volatile emission and plant-insect interaction. Consistently greater quantities of two terpenoids were found in the headspace of potato previously exposed to volatiles from undamaged onion plants identified by mass spectrometry. Using live plants and synthetic blends mimicking exposed and unexposed potato, we tested the olfactory response of winged aphids, *Myzus persicae*. The altered potato volatile profile deterred aphids in laboratory experiments. Further, we show that growing potato together with onion in the field reduces the abundance of winged, host-seeking aphids. Our study broadens the ecological significance of the phenomenon; volatiles carry not only information on whether or not neighbouring plants are under attack, but also information on the emitter plants themselves. In this way responding plants could obtain information on whether the neighbouring plant is a competitive threat and can accordingly adjust their growth towards it. We interpret this as a response in the process of adaptation towards neighbouring plants. Furthermore, these physiological changes in the responding plants have significant ecological impact, as behaviour of aphids was affected. Since herbivore host plants are potentially under constant exposure to these volatiles, our study has major implications for the understanding of how mechanisms within plant communities affect insects. This knowledge could be used to improve plant protection and increase scientific understanding of communication between plants and its impact on other organisms.

## Introduction

Volatile organic compounds (VOCs) released by herbivore damaged plants are involved in a wide range of interactions and play important roles in coexistence between plants and organisms on other trophic levels. They can repel herbivores and attract the herbivore’s natural enemies [1]. They are also involved in rapid defence signalling [2] and neighbouring plants can eavesdrop on them, inducing their own defences and changing their volatile profiles [3].

However, plants release VOCs even when they are not attacked or mechanically damaged, and these volatiles are available as cues for neighbouring plants. Studies have shown that plants can respond to undamaged neighbours via chemical signals [4] and that these responses affect patterns of growth and biomass allocation [5]. Plants are limited in their ability to choose their neighbours but they are able to sense their environment, and volatile cues may be one of several ways in which they gather information about neighbours and respond with appropriate morphological and physiological responses [5], [6]. Plants that grow in high canopy density can also detect neighbours through changes in light quality, which can induce a set of phenotypic traits associated with shade avoidance [7]. Recently it has been shown that volatile chemical exchange between unattacked plants can affect the receiving plant’s interaction with insect herbivores [8]. Thus volatile exchange between plant individuals within stands may affect insect host choice.

It has been shown that intercropping, the practice of growing two or more crops in proximity, can offer advantages in terms of pest control [9], and a range of mechanistic explanations have been proposed to explain the effects on insect colonization and population development [10]–[12]. A role for plant volatiles has been both questioned [13] and supported [14]. However, while direct effects of host volatiles on insects have been considered [15], the possibility that volatile interaction between plants can affect insect host choice through changes in the receiving plant has not been addressed.

The aim of this study was to investigate whether volatile transfer between undamaged plants can contribute to the effects of intercropping on herbivores. We tested this idea in a system consisting of potato (*Solanum tuberosum* L.) intercropped with onion (*Allium cepa* L.) or garlic (*Allium sativum* L.), and the green peach aphid *Myzus persicae* (Sulzer), which uses potato as a host plant. Aphids are an ideal model herbivore since they are major insect pests in many crops and are sensitive to changes in host plant quality [16]. In a field experiment we measured aphid migration into plots of intercropped potatoes and potatoes in pure stands. In laboratory studies we investigated whether exposure of potato to volatiles from neighbouring onion plants influenced aphid olfactory orientation via induced changes in potato volatile emission. Our hypothesis was that volatile exchange between unattacked plants can reduce insect herbivore attraction to an intercrop. We found that exposing potato to VOCs from undamaged onion plants altered its volatile profile and this had a deterrent effect against host-seeking *M. persicae*. In a field experiment, migration of aphids into potato was significantly reduced by intercropping with onion. Our findings represent a novel bottom-up effect of plant co-existence on insect herbivores and provide new evidence of the role of chemically-mediated mechanisms in intercropping.

## Results

### Flight Activity of Aphids in the Field

The emergence of potatoes coincided with the peak of aphid flight because of dry weather conditions after sowing. For this reason the greatest number of *M. persicae* was observed at the first observation occasion and then successively decreased until the middle of July after which aphid flight activity was sporadic.

**Figure 1:**
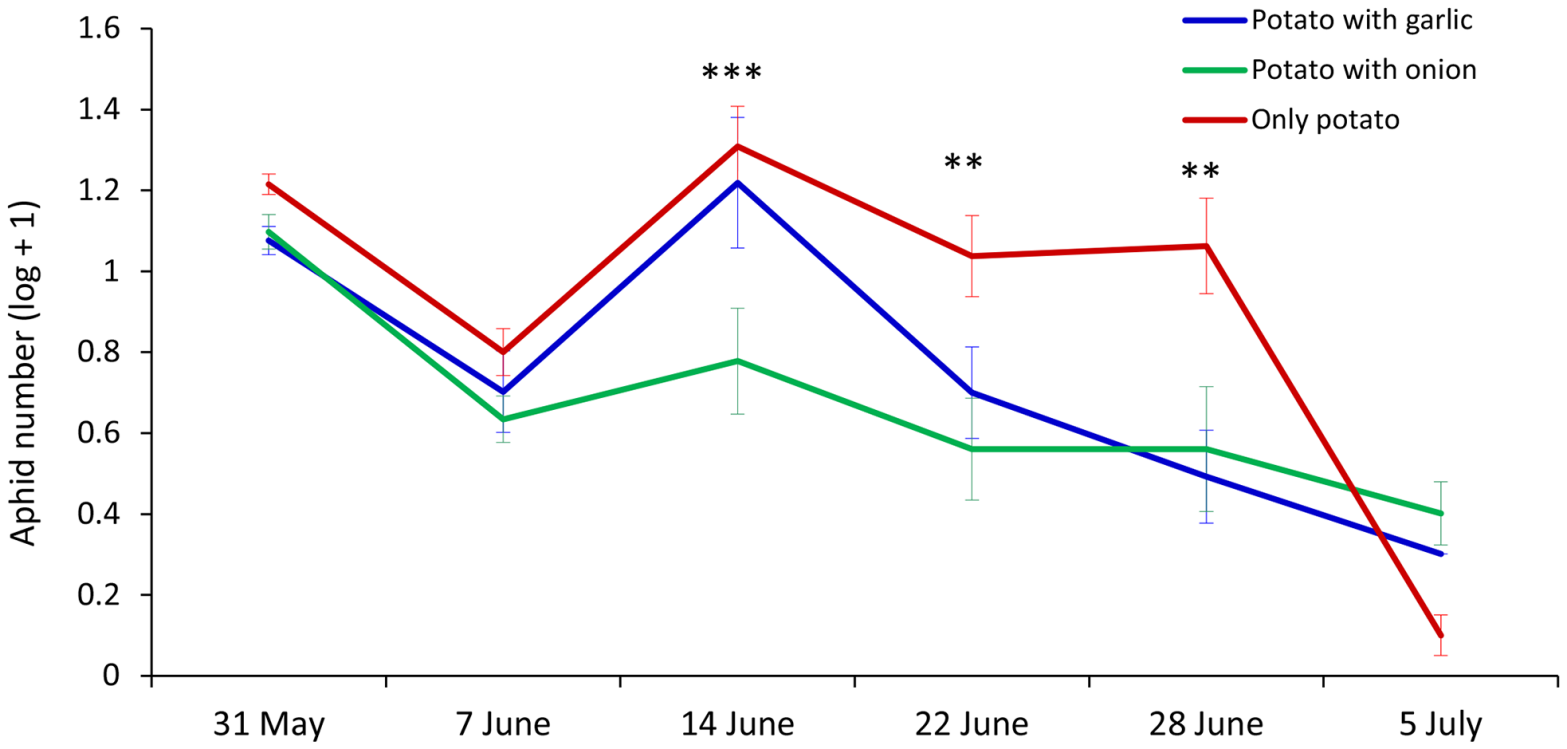
Natural occurrence of winged aphids in intercropped potato with onion and garlic in the field. Mean number of winged *Myzus persicae* caught in yellow water traps in plots with potato in pure stand, potato intercropped with onion, and potato intercropped with garlic, in a field trial in 2009. Data were transformed as natural logarithms. Error bars indicate ± SEM. *P≤0.05; **P≤0.01; ***P≤0.001, Tukey HSD test.

The repeated measurements were best fitted by modelling plots as a random effect according to Akaike’s information criterion. Significant difference in the number of winged aphids caught in the yellow water traps was found between treatments (F_2, 8.72_ = 5.89, P = 0.024). The interaction between treatment (pure and intercropped potato stands) and time was significant (F_10, 23.7_ = 4.15, P = 0.0021), and there were no significant differences between blocks (F_2, 9.31_ = 1.11, P = 0.369). Significantly lower mean numbers of aphids were observed in potato intercropped with onion (P = 0.022, Tukey HSD test) than in plots with only potato plants for the whole experimental period, whereas the number of aphids was not significantly different between plots with garlic and plots with only potato plants (P = 0.105, Tukey HSD test) (Fig. 1).

Numbers of *M. persicae* were significantly lower in potato intercropped with onion compared to pure potato stands on three occasions from the 14^th^ to the 28^th^ of June (P = 0.0009, P = 0.0023, and P = 0.0015, respectively) (Fig. 1). Significant reduction in the number of aphids in potato intercropped with garlic compared with plots with only potato plants was found on the 22^nd^ and 28^th^ of June (P = 0.0247 and P = 0.0004, respectively). Significantly fewer *M. persicae* were found in plots with potato and onion compared to plots with potato and garlic (P = 0.0044) on 14^th^ of June. The results of the field experiments revealed aphid avoidance of potato grown with onion or garlic. Further laboratory experiments were performed with the combination of onion and potato, because of the strong significant effects on the aphid catches in the field with this treatment.

### Aphid Olfactory Responses to Odour from Plants

**Figure 2:**
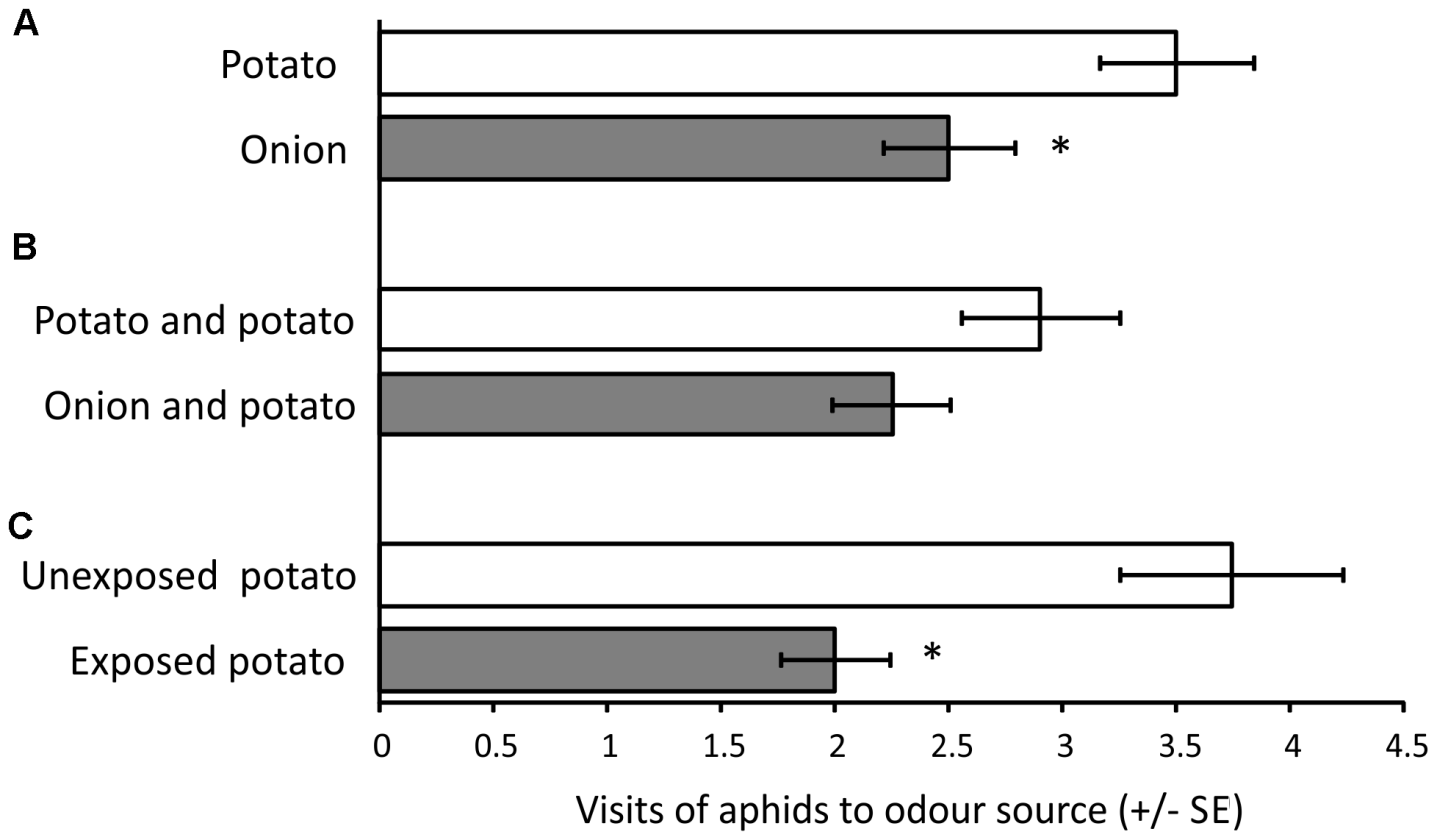
Aphid olfactory responses to volatiles from living plants. Behavioural responses of winged *Myzus persicae* in olfactometer experiments when offered choice between (A) volatiles of onion tested and volatiles of potato, (B) volatile mix of onion and potato and volatile mix of two potato plants, and (C) volatiles of onion-exposed potato and volatiles of unexposed potato plants. Asterisks indicate significant preferences * P≤0. 05, Wilcoxon matched pairs test.

Winged *M. persicae* showed a statistically significant preference for potato plants when given a choice between the odours of onion and potato (Wilcoxon test: Z = 2.430, P = 0.015, n = 16). Aphids showed no difference in response between the mixed odour of onion and potato plants compared with two potato plants (Wilcoxon test: Z = 0.966, P = 0.33, n = 15). Aphids showed a statistically significant preference for the odour of unexposed potato plants compared to the odour of onion-exposed potato plants (Wilcoxon text: Z = 2.414, P = 0.016, n = 15) (Fig. 2).

### Volatile Profile of Onion Plants

The following compounds were identified and quantified in the headspace of onion plants (mean ± SE): 0.53 ng (±0.34) (*Z*)-3-hexen-1-ol, 0.65 ng (±0.22) 1-hexanol, <0.01 ng methyl propyl disulfide, 1.62 ng (±0.31) dimethyl trisulphide, 2.30 ng (±0.84) isopropyl methyl sulphone, 1.31 ng (±0.51) dipropyl disulphide and 7.08 ng (±3.89) 2-undecanone. Accurate quantification of methyl propyl disulfide was not possible due to very low amounts but was less than 0.01 ng.

### Volatile Profile of Potato Exposed to Onion

**Figure 3:**
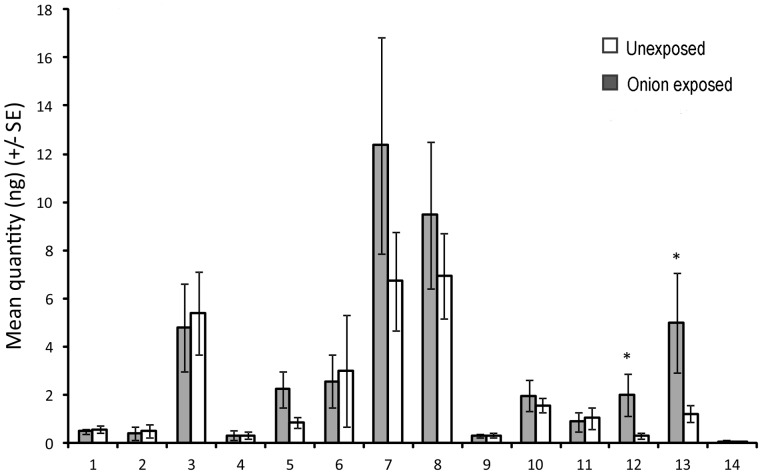
Volatile emissions of onion exposed and unexposed potato plants. Mean quantities (+/− SE) of compounds identified from the headspace of onion-exposed and unexposed potato plants. Compound numbers: 1. (*E*)-2-hexenal; 2. (*Z*)-3-hexen-1-ol; 3. myrcene; 4. limonene; 5. linalool; 6. (*Z*)- 4,8-dimethyl-1,3,7-nonatriene; 7. (*E*)- 4,8-dimethyl-1,3,7-nonatriene; 8. α-copaene; 9. α-cedrene; 10. (*E*)-caryophyllene; 11. (*E*)-β-farnesene; 12. (*E*)-nerolidol; 13. (3*E,7E*)-4,8,12-trimethyl-1,3,7,11-tridecatetraene; 14. 6,10,14-Trimethyl-2-pentadecanone. * P≤0.05 Least Squares Means.

Compounds identified in headspace collections from onion-exposed and unexposed potato plants are shown in Fig. 3. All identifications achieved by mass spectrometry were confirmed by comparison of retention times with those of authentic standards, with the exception of (*Z*)-4, 8-dimethyl-1, 3, 7-nonatriene and 6, 10, 14-trimethyl-2-pentadecanone, for which authentic standards could not be obtained. Canonical analysis of principal coordinates revealed significant differences in overall blend composition between onion-exposed and unexposed potato (P = 0.04). Differences were attributed to the C_15_ sesquiterpene (*E*)-nerolidol (P* = *0.039) and the C_16_ homoterpene (3*E, 7E*)-4, 8, 12-trimethyl-1, 3, 7, 11-tridecatetraene (TMTT) (P* = *0.038), which were both collected in greater quantities from onion-exposed plants compared to unexposed plants.

### Aphid Olfactory Responses to Synthetic Blends and Single Volatile Compounds

**Figure 4:**
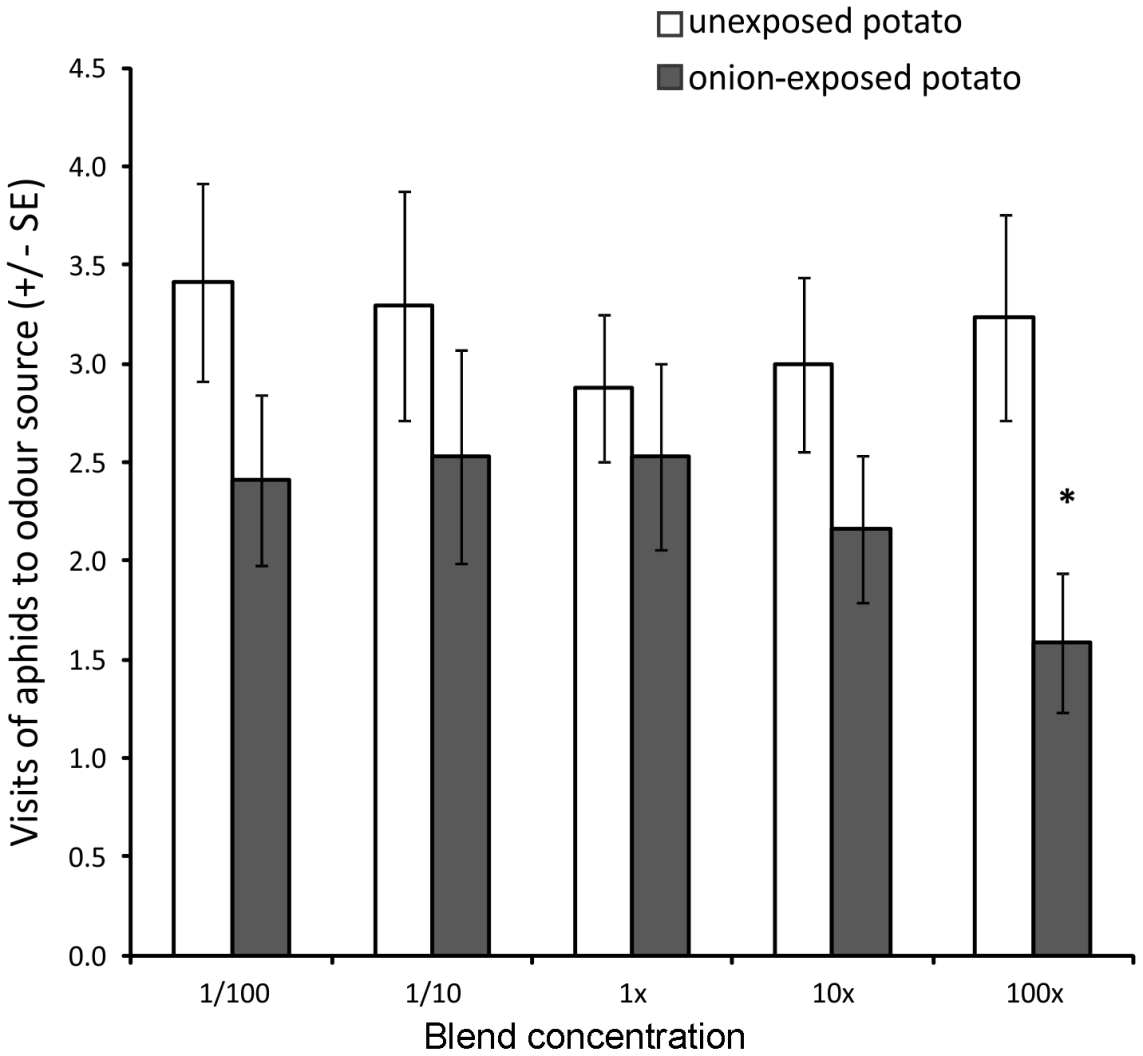
Aphid olfactory responses to synthetic blends of volatile organic compounds of exposed and unexposed potato. Responses of winged *Myzus persicae* in olfactometer experiments when presented with synthetic blends based on headspace collections of onion-exposed (treatment) and unexposed potato (control). Synthetic blends were at 1/100, 1/10, 1x, 10x or 100x the original concentration of volatiles identified in potato headspace. Error bars indicate ± SEM. * P≤0.05 Wilcoxon test.

Winged aphids showed a clear ability to discriminate between the synthetic blends of onion-exposed and unexposed potato plants. Aphids made significantly fewer visits to the olfactometer arm containing the synthetic blend of onion-exposed potato than to the arm containing the blend representing unexposed potato (Wilcoxon test: Z = 1.988, P = 0.047, n = 15), but only at the highest concentration tested (Fig. 4).

**Figure 5:**
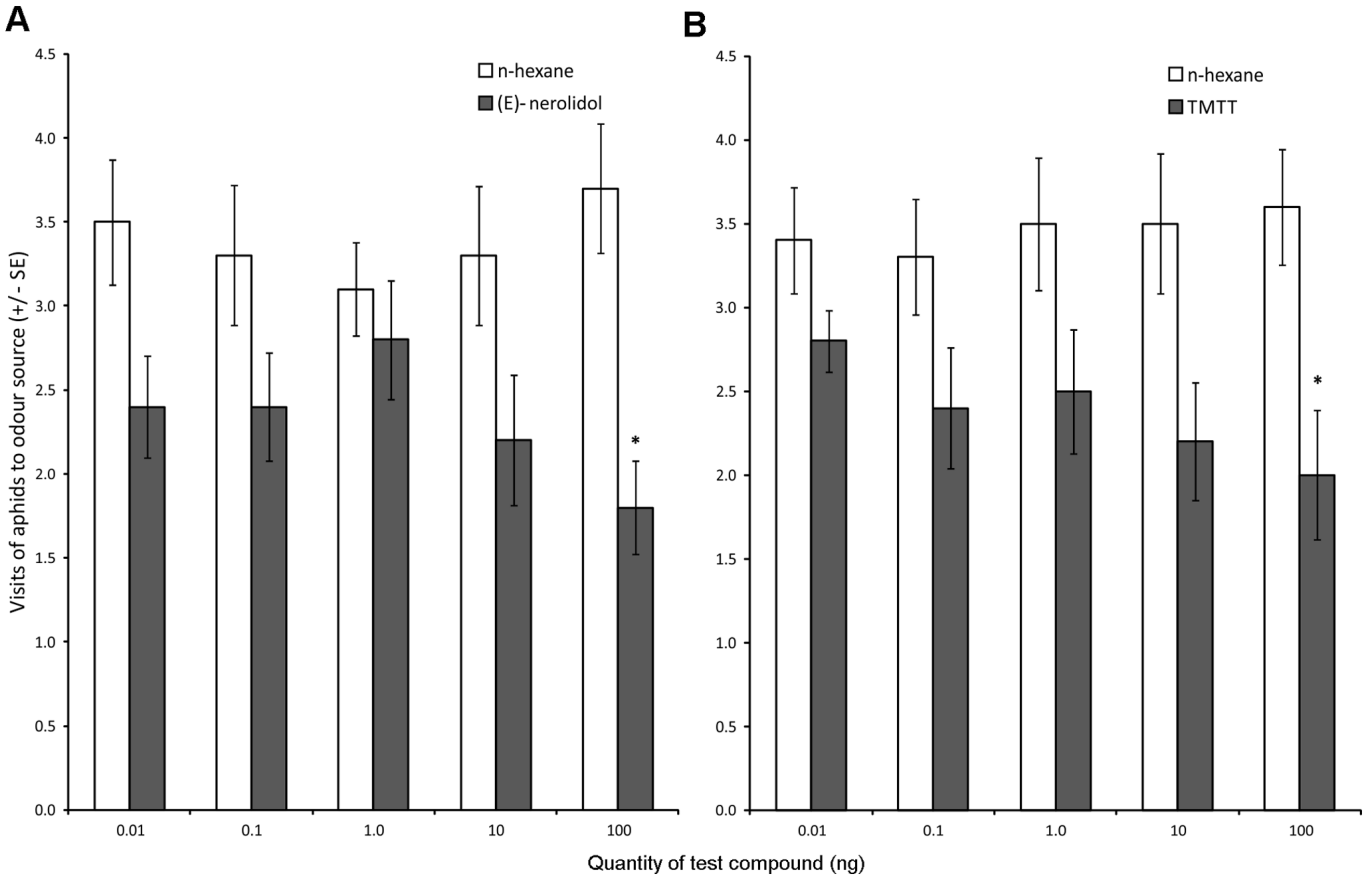
Aphid olfactory responses to two terpenoids realised in greater quantities from potato exposed to onion. Responses of winged *Myzus persicae* in olfactometer experiments when presented with test solutions containing different doses of (A) (E)-nerolidol, and (B) (3E, 7E) 4, 8, 12-trimethyl-1, 3, 7, 11-tridecatetraene (TMTT), alongside hexane control. Error bars indicate ± SEM. *P≤0.05 Wilcoxon test.

Figure 5 shows the behavioural responses of *M. persicae* to each compound presented individually at each dose tested. Aphids visited the treated region of the olfactometer significantly less when a 100 ng µl^-1^ dose of (E)-nerolidol (Wilcoxon test: Z = 2.77, P = 0.0056, n = 17) or 100 ng µl^-1^ of TMTT (Wilcoxon test: Z = 2.21, P = 0.027, n = 23) were used as odour sources.

## Discussion

Exposure of potato plants to VOCs from undamaged onion plants significantly alters their volatile profile leading to avoidance by aphids in laboratory experiments. In the field, the abundance of winged, host-seeking aphids was lower in a potato-onion intercrop than in potato grown in pure stand. Volatile exchange between undamaged plants may represent a novel mechanism contributing to the observed effects of botanical biodiversity on insect herbivores.

### Interaction between Undamaged Plants by Volatiles Modifies Volatile Profiles of Responding Plants

Our results show that the headspace of potato plants previously exposed to volatiles from onion contained approximately four times greater concentrations of the terpenoids (*E*)-nerolidol and TMTT compared to the headspace of unexposed plants. This is the first report of a change in the volatile profile of plants induced by volatile interaction with undamaged plants. Such induction has previously been shown to occur in response to volatiles released from herbivore-attacked plants [17], [18]. This has been interpreted as a means by which plants can obtain early warning of herbivore presence in their immediate environment. Our results imply that VOCs carry not only information on whether neighbouring plants are under attack, but also on the emitter plants themselves. In this way responding plants could assess whether the neighbouring plant is a competitive threat and adjust their growth accordingly. We interpret this as an adaptation towards future competition. It has been shown that plants respond to volatiles from neighbouring plants with morphological changes that may prepare them for competition [5]. Since neighbouring plants are likely to compete for resources, the detection and response to the presence of a potential competitor should benefit plants [19]. Further, the changes in volatile profile of responding plants indicates that volatile cues from neighbouring plants induce physiological changes in responding plants, which have significant effects on insect herbivores.

Plant volatiles can be passively adsorbed and re-released by neighbouring plants, reducing herbivore presence [20]. Chemical analysis in our study showed that the terpenoids released in higher amounts by onion-exposed potato plants were not detected in the headspace of onion. Only one compound detected from onion was also detected in potato, (*Z*)-3-hexen-1-ol, and it was not enhanced after exposure. It is possible that onion-exposed potato absorbed and released traces of onion volatiles that were below the detection limits of our analyses. However, in the olfactometer, aphids did not discriminate between the odours of onion and potato combined and potato alone, suggesting that adding onion volatiles to the potato headspace does not affect aphids. Thus, while we cannot conclusively rule out the absorption of trace amounts of onion volatiles onto potato, associational resistance via passive absorption is unlikely to explain our results.

Plants are able to sense changes in their environment and adjust their morphology, physiology and phenotype accordingly [21], [22]. There are a number of stimuli that plants perceive and can react to: chemicals, temperature, light, moisture, gravity, pathogens, physical disruption and touch [23]–[28]. Reduction in the red to far-red ratio of canopy light can be used by plants as a warning signal of future competition [7], however in our study, observed changes in aphid migration in the field occurred at the early seedling stage of plants, so plant responses to shading are unlikely to have contributed to the changed aphid behaviour in the field. Plants are able to detect VOCs released from herbivore damage plants and these herbivore-induced VOCs play important roles in interactions between plants and arthropods [17], [29], [30]. Many studies have shown that intact plants growing in the neighbourhood of a damaged VOC-releasing plant respond to these chemical cues with biochemical changes [18], [31], [32]. Neighbouring plants can respond to VOCs by changing transcription patterns of defence-related genes [33], and they may increase the production of hormones and other VOCs [31]. This phenomenon has been defined as a prophylactic reaction toward future herbivore attack [34]. Our study shows for the first time that plants also respond to VOCs from undamaged neighbours, broadening the potential ecological significance of plant-plant chemical interaction. If these adaptations involve changes in plant physiology, then herbivores that are sensitive to host quality, such as winged aphids, may be able to detect and respond to them. This represents a novel mechanism by which the structure of plant communities can affect insect herbivores.

### Aphid Olfactory Responses to Synthetic Volatile Blends and Terpenoids

Aphids rely heavily on olfaction when searching for a suitable host [16]. Winged aphid morphs undertake the first stages of host location, selection and population establishment, using host volatile cues. The use of winged morphs in our olfactory bioassays indicates how changes in potato volatile emission may affect orientation of host-seeking aphids in the field. Aphids preferred the odour of unexposed potato over the odour of onion-exposed potato, suggesting a behavioural response to the changes in volatile emission induced by the exposure. The two terpenoids released in higher amounts by exposed potato, TMTT and (*E*)-nerolidol, were both significantly repellent to aphids at the highest dose tested, which could partly explain the reduced attraction to onion-exposed potato in the olfactometer. The homoterpene TMTT is widely reported as a herbivore-induced volatile and inter-plant signal [35] and has been shown to repel aphids [36]. (*E*)-nerolidol is the precursor of DMNT, a sesquiterpene released from many plant species after herbivory and an active component in mediating interplant signal transfer [37]. We detected greater amounts of DMNT in the headspace of onion-exposed potato than in unexposed potato, but this difference was not statistically significant.

We constructed synthetic volatile blends based on headspace from onion-exposed and unexposed potato. Behavioural responses to these blends reflected similar responses to the odour of living plants. Following this, we tested responses to the two volatiles that were significantly increased in onion-exposed potato. Aphids avoided these compounds in the olfactometer, suggesting that increased emission of these may be at least partly responsible for reduced attractiveness of onion-exposed potato. It is interesting that winged aphids only responded to the highest doses of TMTT and (*E*)-neroldiol in olfactometer assays. Previous studies with aphids have shown that behavioural responses to volatiles may only occur at higher concentrations, similar to those tested in the present study [38], [39]. The reason for this is largely unknown but it is possible that limited sensitivity of the olfactory sense means that aphids have difficulty responding to lower concentrations of volatiles.

### Flight Activity of Aphids in the Field

Insect herbivores have been found to be less abundant in intercropped systems compared to monocultures [40]. The results of our field experiment indicate selective aphid migration to potato plants in pure stands and our olfactory results showed that aphids preferred volatiles from their host-plant potato over onion, a non-host. This is in line with field studies in which intercropping with *Allium* species reduced populations of *M. persicae* in a potato crop [41], [42]. Earlier studies speculated that intercropped plants might mask olfactory and visual cues used by herbivores to find their host, or confuse or repel the insects [13], [15], [43]. However, a mixture of onion and potato odour was not less attractive than potato odour in our olfactory study, suggesting that onion did not mask the odour of potato for *M. persicae.* Aphids showed significant negative responses to the odour of potato plants that were previously exposed to onion volatiles. The laboratory results suggest a mechanism based on volatile exchange between plants rather than odour masking. We suggest that the reduction in abundance of winged aphids in intercropped plots in the field is consistent with the mechanistic explanation proposed from the results of our laboratory experiments, and that our study supports a prominent role for plant volatile cues in intercropping.

We present chemical and behavioural evidence from laboratory and field experiments showing that volatile interactions between undamaged plants can induce volatile emissions in the exposed plant, in turn influencing host location by insect herbivores. Similar effects have been found in barley exposed to VOCs from weeds [4], [44], [45] or other barley cultivars [46], [47], which resulted in reduced aphid performance on volatile-exposed plants. To what extent plants in general are able to differentially perceive and respond to their neighbours in the way described here remains to be determined. In the present study we have shown that exposure to plant volatiles can result in differences in volatile emission in the exposed plant, which has not been shown previously and may partly explain reduced aphid performance in earlier studies. If it is found to be widespread, this mechanism could have major implications for the study of plant-plant and plant-insect interactions. This previously unknown interaction may affect orientation of host-seeking insects in the field, contributing a new potential mechanism to the discussion on how and why biological diversification can reduce pest insect populations. Using this knowledge, it may be possible to develop novel crop protection strategies by engineering or selecting crop plants with altered volatile production.

## Materials and Methods

### Plants and Aphids for Laboratory Experiments

Sprouting buds of potato tubers, *S. tuberosum* were cut and planted individually in plastic pots (8×8×8 cm) with potting soil (Special Hasselfors garden, Hasselfors, Sweden). One bulb of onion, *A. cepa* was planted per pot. Plants were produced in a greenhouse maintained at 18–22°C with a light regime of L16:D8. Natural light was supplemented by light from HQIE lamps. To prevent plant-plant interaction during the pre-experimental period, onion and potato plants were produced in separate greenhouse chambers. Potato tubers (cv. Sava) were obtained from Lantmännen, Sweden. Onion bulbs (cv. Stuttgarter Riesen) were provided by Weibulls Horto, Sweden. The same varieties of each species were used for field and laboratory experiments. To avoid effects of damage-related VOCs, only visibly undamaged plants were used.

Green peach aphids, *M. persicae* (Sulzer), derived from a stock culture maintained on potted rapeseed plants (*Brassica napus* L.) grown under similar conditions as the test-plants, were used for all experiments. Adult alatae (winged) individuals of *M. persicae* were used for all experiments.

### Field Experiments

A field experiment was conducted at Radmilovac (44°76′N, 20°58′E), Serbia. A Latin square design was used, with 9 plots (5 m×5 m) randomly repeated in each of three blocks. The distance between the plots was 1 m. Three treatments were compared in each block: potato in pure stands, potato intercropped with onion, and potato intercropped with garlic. The row spacing was 70 cm and the distance between potato plants in a row was 40 cm. Garlic and onion bulbs were planted between potatoes in the rows with the same distance between potato plants as in pure stand, which is common practice in region. To decrease edge effects on insect movement, a ten-meter area of potato (cv. Aladin) was planted around the experimental field. No pesticides were used, and weeds and insect herbivores other than *M. persicae* were manually removed. Yellow water traps (17×17×10 cm) containing water with 1% detergent, were placed on the ground in the middle of each treatment. During the growth of the crop, the traps were successively raised in height to remain visible to aphids. Samples were taken once per week and the captured insects were kept in 70% alcohol until identification. For identification of aphid species, a binocular loupe and keys for identification of winged aphids were used [48].

To compare estimates of aphid immigration made repeatedly at intervals in the same plots during the experimental period, mixed linear models were used as suggested by Fitzmaurice [49] and Littell [50]. The number of aphids observed per plot was expressed as log (aphid number +1). The dependence between observations over time was modelled using a spatial power covariance matrix. The models included the fixed effect of treatment, block, time and the treatment by time interaction. Interaction between blocks and time points was included in the model as an independent factor, normally distributed random effect. Least squares means were calculated and compared using Tukey HSD test. Diagnostic plots were used to diagnose the models for normality and homoscedasticity. The Mixed procedure of the SAS package [51] was used for the analyses. No permits for field experiments were required for the described study, the land accessed for the field experiment in Serbia is owned by the Faculty of Agriculture in Belgrade, and no protected species were sampled.

### Laboratory Experiments

#### Exposure of plants to volatiles

Exposure of one plant to volatiles from another was done in a series of two-chamber cage experiments [5]. The exposure system consisted of a series of clear Perspex cages divided into two chambers - inducing and responding (each 10×10×40 cm), connected by an opening (7 cm diameter) in the middle wall. Air entered into the system through the chamber with an inducing onion plant, passed through the hole in the middle wall into the chamber with a responding potato plant and was removed from the greenhouse by a fan. Control treatments consisted of two-chamber cages with potato plants in the responding chamber and an empty inducing chamber. Airflow through the system was 1.3 l min^-1^. Individual pots were watered using an automated drop system (DGT Volmatic) without additional fertilizer, and were placed in separate Petri dishes in the chambers preventing interactions between plants by root exudates. The two-chamber cages were kept in a greenhouse at 18–22°C and a light regime of L16:D8. The exposure time was five days, based on previous studies of volatile interaction between plants [45]. Immediately after exposure, plants were used for olfactometer studies.

#### Olfactory bioassays

The results of the field experiments showed aphid avoidance of potato grown with onion and garlic respectively. Subsequent olfactometer experiments were designed to examine whether olfactory orientation may contribute to the pattern observed in the field. The combination of onion and potato was chosen for mechanistic studies because it gave the strongest significant effects on aphid catches in the field. Olfactory responses of aphids were measured using a two-way airflow olfactometer consisting of two stimulus zones (arms) directly opposite each other, with a central neutral zone separating them [4]. Air was drawn from the centre of the olfactometer using a vacuum pump, establishing discrete air currents in the side arms. Airflow in the olfactometer was set to 180 ml/min, measured with a flow meter at the arm inlets.

When plants were used as odour sources, the two-chamber cages containing the plants were connected directly to the arms of the olfactometer. Three different treatment arrangements were designed: a) an unexposed potato plant was tested against an onion plant, b) an unexposed potato and an onion plant were tested against two unexposed potato plants, and c) a potato plant that had been previously exposed to an onion plant was tested against an unexposed potato plant. When two plants were used on each side of the olfactometer in b), each was contained in a separate cage and the two connected to the inlet of the olfactometer using Y-connectors. In this way the olfactometer contained volatiles from both plants without the plants interacting.

To confirm behavioural differences in the response of aphids to the odour of onion exposed- and unexposed potato plants, dose-response olfactometer experiments were conducted using serial dilutions of synthetic blends based on volatiles quantified in the plant headspace. Chemicals for the blends were obtained as described under ‘chemical analysis’ below. The quantitative and qualitative composition constructed blends was confirmed by GC-FID and GC-MS. As a starting point, synthetic blends were constructed comprising all identified compounds in the same ratio as in the headspace collections, but with each compound at 10x the concentration (Fig. 3). Serial dilutions were then made of each concentrated blend, giving five test blends of different concentrations for both onion-exposed and unexposed plants: 1/1000, 1/100, 1/10, 1x and 10x the concentration of volatiles collected from the headspace (Fig. 2). Aphid olfactory response to the synthetic blend of potatoes previously exposed to volatiles from onion plants was tested against the synthetic blend of unexposed potatoes. Test blends were dosed at a volume of 10 µl on small pieces of filter paper, allowed to evaporate for 30 s and placed into glass tubes (2.5 mm diameter) connected to holes in the sides of the olfactometer arms. Since 10 µl of test solution were used, test concentrations in the olfactometer were 1/100, 1/10, 1x, 10x and 100x the concentration of volatiles collected from plants.

To confirm differences in the behavioural responses of aphids to the synthetic blends, dose-response olfactometer experiments were conducted with the two compounds that were significantly more abundant in the headspace of onion-exposed potatoes compared to unexposed potatoes (Fig. 2). (*E*)-nerolidol and (3*E*, 7E)-4, 8, 12-trimethyl-1, 3, 7, 11-tridecatetraene (TMTT) were tested against redistilled n-hexane at five different concentrations: 0.01 ng, 0.1 ng, 1 ng, 10 ng, and 100 ng.

Winged aphids were randomly chosen from the cultures, using a fine paintbrush and placed in Petri dishes with moistened filter paper to prevent dehydration. Aphids were left in the bioassay room for at least 2 h to acclimatize prior to experiments. A single aphid was introduced into the olfactometer through a hole in the top. After an adaptation period of 10 minutes, the position of the aphid in the arena was recorded at three minute intervals over a 30 minute period. The accumulated number of visits of a single aphid in the arms with the different odour sources was regarded as one replicate. Pseudo replication was avoided by using a single aphid in each replicate, testing each aphid only once, and by using a clean olfactometer for each replicate. The number of replications was between 16 and 28. The test was terminated if an aphid did not move for longer than 10 minutes and these individuals were not included in the analysis. Before each test insect, olfactometers were rotated 180° to avoid positional bias.

For comparisons of the number of aphid visits in the control and the treated arm, Wilcoxon matched pairs test was used. Results showing p-values at the 5% level were considered to be significant. Statistical analyses were performed with Statistica software version 10 [52].

#### Volatile collection

Air entrainment was used to collect volatiles from the headspace of onion plants and of onion-exposed and unexposed potato plants. Prior to entrainment, polyethylene terephthalate (PET) oven bags (Toppits®, Melitta Scandinavian AB, Sweden), aluminium foil and Teflon materials were baked in an oven at 140°C for at least 2 h. Charcoal filters were baked out similarly under a flow of nitrogen, and Tenax tubes were heated at 220°C under a flow of nitrogen for at least 2 h to remove contaminants.

Exposure of potato plants to onion volatiles was carried out as described above during for days. After exposure, plants were transferred to a controlled environment room (21°C). Each pot was covered with aluminium foil which covered the soil and individually enclosed in 60×55 cm PET oven bags, sealed around the pot with rubber bands. Pots of soil were entrained as a control. Teflon tubing was inserted under the rubber band and charcoal-filtered air pumped in at a rate of 600 ml min^-1^. A small hole was cut in the top corner of the bag and a glass tube (80 mm×3 mm i.d. containing 0.05 g Tenax TA 60/80 mesh, Supelco Inc., Bellefonte, P.A.) was inserted. The Tenax tube was connected to a pump via a brass fitting and Teflon tubing, and air was drawn through the tube at a rate of 400 ml min^-1^. The difference in flow rates created a positive pressure preventing contaminated air from entering. The rubber bands did not create an air-tight seal so there was no continuous build-up of air pressure inside the system. Air was pumped in for one hour prior to volatile collection in order to flush any contaminating volatiles. Volatile collection was carried out for 24 h. After entrainments had finished, 50 µl of 1 ng µl^-1^ 2-tridecanone was injected onto each Tenax tube as an internal standard before being sealed with nitrogen in a glass ampoule and stored at -20°C until analysis. Entrainments were replicated nine times for onion-exposed potato plants, eight times for unexposed potato plants, six times for onion plants and four times for the control pots of soil. Two additional entrainments of each were carried out for chemical identification using coupled gas chromatography-mass spectrometry.

#### Chemical analysis

Separation of volatiles was carried out on a nonpolar HP-1 bonded-phase fused silica capillary column (50 m × 0.32 mm i.d., film thickness 0.52 µm) housed in a Hewlett-Packard 6890N GC equipped with a flame ionization detector (FID). Volatiles on the Tenax were transferred to the GC column by thermal desorption using an Optic 3 programmed temperature vaporization inlet (Atlas GL int.), set for rapid heating from 30°C to 220°C at 16°C sec^-1^. The oven temperature was maintained at 30°C for 2 min, then programmed at 5°C min^−1^ to 150°C and held for 0.1 min, then 10°C min^−1^ to 250°C. The carrier gas was hydrogen. GC traces for each of the entrainment samples were compared with traces of the pot of soil controls to highlight any peaks corresponding to compounds collected from the plants. The areas of these peaks were quantified using the peak corresponding to the internal standard. Canonical analysis of principal coordinates (CAP) was used to determine if there were overall differences between blends from onion-exposed and unexposed potato [53]–, [54]. When a significant difference was found, one-way ANOVA with Least Squares Means post hoc analysis on arcsinh transformed data was used to determine significant differences between individual compounds.

Identification of compounds was achieved by gas chromatography-mass spectrometry. Volatiles were removed from the Tenax by thermal desorption as described previously. Volatile separation was achieved on a non-polar column (50 m × 0.32 mm i.d. HP1) housed in an Agilent 7890A GC that was directly coupled to a mass spectrometer (Agilent 5975 C inert MSD with triple-axis detector). Ionization was by electron impact at 70 eV. Oven temperature was maintained at 30°C for 2 min and then programmed at 5°C min^−1^ to 150°C, where it was held for 0.1 min, then 10°C min^−1^ to 250°C. Identifications were made by comparison of spectra with those in a commercially available library (NIST 2008) and confirmed by comparing retention times with those of authentic standards. For bioassays and compound confirmations, chemical standards were purchased at the following purities from Sigma-Aldrich (Sweden) unless otherwise stated: (E)-2-hexenal (98%), (*Z*)-3-hexen-1-ol (98%), 1-hexanol (>99%), myrcene (Fluka, >95%), limonene (90%), linalool (97%), α-copaene (90%), α-cedrene (>95%), (E)-caryophyllene (Fluka, 99%), (E)-β-farnesene (Fluka >90%), (E)-nerolidol (Fluka >85%), 2-undecanone (99%), methyl propyl disulfide (90%), dimethyl trisulfide (>99%), dipropyl disulfide (98%), isopropyl methyl sulfone (TCI Europe, Belgium, >96%). (E)- 4,8-dimethyl-1,3,7-nonatriene and (3E,7E)-4,8,12-trimethyl-1,3,7,11-tridecatetraene were provided by Dr Michael Birkett, Rothamsted Research. Standards of (Z)- 4,8-dimethyl-1,3,7-nonatriene and 6,10,14-trimethyl-2-pentadecanone were unavailable.
